# RNPC1 enhances progesterone receptor functions by regulating its mRNA stability in breast cancer

**DOI:** 10.18632/oncotarget.12016

**Published:** 2016-09-14

**Authors:** Peipei Lou, Chunlian Li, Liang Shi, Tian-Song Xia, Wenbin Zhou, Jing Wu, Xujie Zhou, Xiaoxia Li, Ying Wang, Ji-Fu Wei, Qiang Ding

**Affiliations:** ^1^ Jiangsu Breast Disease Center, The First Affiliated Hospital with Nanjing Medical University, Nanjing City, Jiangsu Province, 210000, China; ^2^ Research Division of Clinical Pharmacology, The First Affiliated Hospital with Nanjing Medical University, Nanjing City, Jiangsu Province, 210000, China

**Keywords:** progesterone receptor, RNPC1, mRNA stability, breast cancer, proliferation

## Abstract

Progesterone receptor (PR) could activate transcriptional process involved in normal mammary gland proliferation and breast cancer development. Moreover, PR expression is an important marker of luminal breast cancer, which is associated with good prognosis and indicates better responding to endocrine therapies. The regulation of PR expression was studied mainly on its post-translational levels. In this study, we found PR was positively regulated by RNA-binding region-containing protein 1 (RNPC1), a RNA-binding protein, in PR positive breast cancer. Overexpression of RNPC1 increased, whereas knockdown of RNPC1 decreased, the level of PR protein and transcripts. Additionally, we demonstrated that RNPC1 could bind to PR mRNA via AU-rich elements (AREs) within PR 3′-untranslated region (3′-UTR) and then enhance PR mRNA stability. Moreover, we proved that progesterone-dependent PR functions which could induce breast cancer proliferation were enhanced by RNPC1, both *in vitro* and *in vivo*. Conclusively, we revealed a novel mechanism by which PR could be regulated by RNPC1 via stabilizing its mRNA.

## INTRODUCTION

Breast cancer is a complex disease, which involves multiple risk factors. Among these risk factors, reproductive history and menstrual state are relevant with ovarian steroid hormones [1–4]. For women, the major ovaries steroid hormones are estrogen and progesterone, which are both responsible for development of female sex characteristics and maintenance of menstrual cycle. Besides, long-term exposure of elevated levels of ovaries steroid hormones are known to increase the breast cancer risk in pre-menopausal women [3, 5].

Among the ovaries steroid hormones, progesterone exhibits its mitogenic effects predominantly by direct action or paracrine signaling on the mammary gland [6, 7]. The physiological actions of progesterone are modified by interacting with its receptor, progesterone receptor (PR), one of the ligand activated transcription factor [8].

PR activates the progression associated with mammary gland proliferation and breast cancer by binding to DNA either through cis-acting progesterone response elements (PREs) directly or by binding to other DNA bound transcription factors indirectly [9–12]. In addition to its action as transcription factor, PR can migrate outside of the nucleus and mediate rapid progesterone-induced activation of a series of alternative, non-genomic protein phosphorylation signaling cascades [13–15]. Furthermore, non-genomic activation by PR in turn leads to phosphorylation of PR, which promotes binding to its PREs or other elements on target DNA through tethering interactions with other transcription factors [16].

In clinic, PR-positive breast cancers respond better to selective ER modulator (SERM) therapy, and had a significant improvement than PR-negative tumors [17, 18]. PR also has a tight relationship with disease-free survival and overall survival in breast cancer [17, 19]. A recent study defined luminal A tumors, more than 20% of PR positive tumor cells predicted significantly better survival, which was added to St Gallen guideline using PR threshold of 20% to differentiate Luminal A from Luminal B breast cancer [20, 21]. Therefore, based on the expression of ER, PR, HER2 and Ki-67 detected by immunohistochemistry (IHC), breast cancer is classified into five groups: luminal A (ER and PR-positive/HER2 negative/Ki-67 ‘low’), HER2-negative luminal B (ER positive/HER2-negative/Ki-67 ‘high’ or PR ‘negative or low’), HER2-positive luminal B (ER-positive/HER2 overexpression/any Ki-67 or PR), HER2-positive (HER2 overexpression/ER and PR absent) and triple negative (ER and PR absent/HER2-negative) [21]. Determination of ER and PR expression by IHC test is essential for clinical application and routinely used to predict the prognosis and to estimate patients who are most likely to benefit from endocrine therapy.

In fact, PR expression was regulated exquisitely in breast cancer. Previous studies mainly focused PR expression regulation on its post-transcriptional modification, which included phosphorylation, acetylation, ubiquitylation, and sumoylation [22]. For example, phosphorylation of PR with progestin treatment regulated both their ligand-dependent and ligand-independent transcriptional activities [23, 24]. Additionally, acetylation of PR could negatively regulate transcriptional activities of PR [24]. Ubiquitylation of PR, which participated in PR degradation, contradictorily increased their transcriptional activity [25]. Post-translational modification by small ubiquitin-related modifiers (SUMO) appeared to play multiple roles in PR regulation, including targeting, stabilization, and transcriptional activation [23, 26]. However, regulation of PR in mRNA level was rarely reported. In the present study, we presented a unique mechanism for the regulation of PR expression by stabilizing its mRNA. RNA-binding region-containing protein 1, a RNA binding protein, could bind to PR mRNA via the AU-rich elements (AREs) within PR 3′-untranslated region (3′-UTR) and then enhance PR mRNA stability. Moreover, the progesterone-dependent PR functions which induce breast cancer proliferation could be enhanced by RNPC1, both *in vitro* and *in vivo*.

## RESULTS

### IHC staining of RNPC1 and PR in human breast cancer tissues

**Figure 1:**
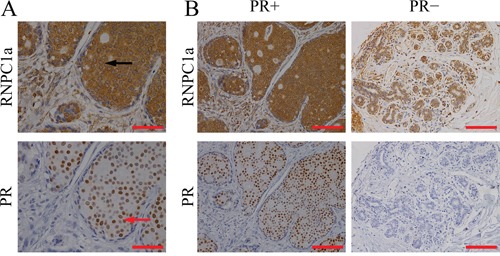
RNPC1 expression correlated with PR in breast cancer tissues **A**. IHC analysis of RNPC1a and PR in breast cancer at 200× magnification. RNPC1a was mainly expressed in the cytoplasm (black arrowed) and PR was mainly expressed in the nucleus (red arrowed). Scale bars indicated 200 μm. **B**. IHC analysis of RNPC1a and PR in breast cancer at 400× magnification. PR positive breast cancer expressed high level of RNPC1a; PR negative breast cancer expressed low level of RNPC1a. Scale bars indicated 100 μm.

RNPC1 is expressed as two isoforms, while RNPC1a with 239 amino is the largest isoform of RNPC1 [27]. IHC staining was used to examine whether there was any correlation of location and expression between RNPC1a and PR in 90 breast cancer tissue. RNPC1a was mainly expressed in the cytoplasm and PR was mainly expressed in the nucleus (Figure 1A). The representative images of RNPC1a expression in PR positive and negative breast cancer tissues were showed in Figure 1B. It indicated that RNPC1a expression was significantly correlated with PR in breast cancer (p<0.05). The correlation between RNPC1a expression and clinic pathological features was showed in Table 1. RNPC1a and PR cellular localization in breast cancer cells was confirmed using immunofluorescence (Supplementary Figure S1).

**Table 1:** Assiociation of RNPC1a with PR and clinicopathological characteristics of breast cancer

ClinicopathologicalCharacteristics	RNPC1a expression			
	No. of case	Low(%)	High(%)	*P*-value
Age				0.200
<50	49	35(71.4)	14(28.6)	
≥50	41	24(58.5)	17(41.5)	
Pathological grade				0.328
I-II	67	42(62.7)	25(37.3)	
III	23	17(73.9)	6(26.1)	
TNM stage				0.255
I-II	82	52(63.4)	30(36.6)	
III	8	7(87.5)	1(12.5)	
PR				0.028
negative	60	44(73.3)	16(26.7)	
positive	30	15(50)	15(50)	

### RNPC1a regulated PR expression in PR positive breast cancer cells

To verify endogenous RNPC1a could regulate PR expression, RNPC1a was overexpressed and knocked down in MCF-7, BT474, MDA-MB-231 and SUM 1315 cells. PR expression had no change in MDA-MB-231 and SUM 1315 cells (Supplementary Figure S2). It indicated that RNPC1a could not alter PR expression in PR negative breast cancers.

**Figure 2:**
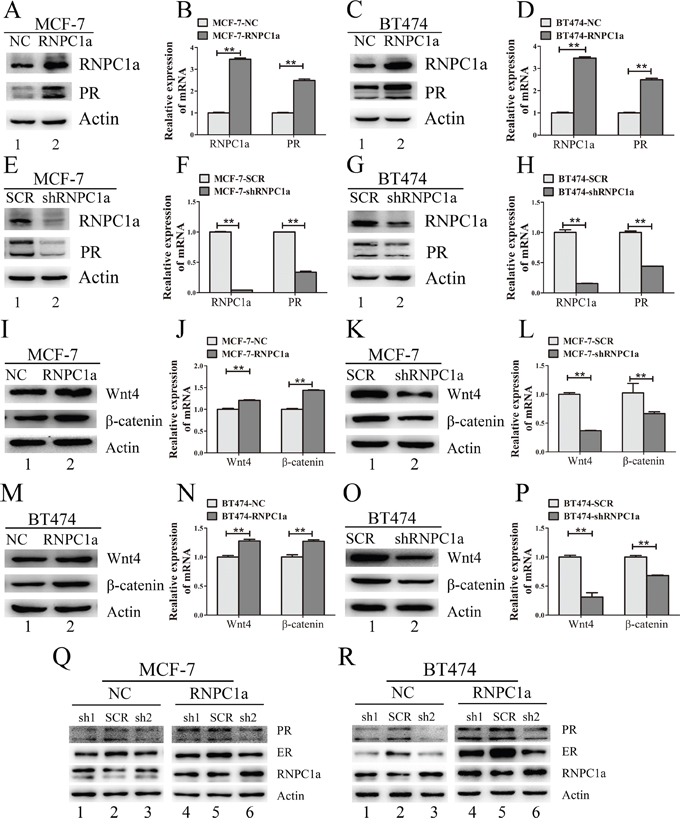
PR and its downstream gene was regulated by expression of RNPC1 in PR positive breast cancer cells **A-D**. MCF-7 and BT474 cells were transfected with lentivirus to overexpress RNPC1a (RNPC1a) and the control (NC). PR expression was obviously increased after RNPC1a up-regulated both in protein (A, C) and RNA levels (B, D). **E-H**. MCF-7 and BT474 cells were transfected with lentivirus to knockdown RNPC1a (shRNPC1a) and the control (SCR). PR expression was obviously decreased after RNPC1a down-regulated both in protein (E, G) and RNA levels (F, H). Western blot and qRT-PCR were applied to detect the expression of RNPC1a and PR. **I-P**. PR target gene expression while RNPC1a was overexpressed or knocked down. (I, J) MCF-7 cells were transfected with lentivirus to overexpress RNPC1a (RNPC1a) and the control (NC). Wnt4 and β-catenin protein (I) and RNA level (J) were increased while RNPC1 was overexpressed. The same effects were also observed in BT474 cells (M, N). (K, L) MCF-7 cells were transfected with lentivirus to knock down RNPC1a (shRNPC1a) and the control (SCR). Wnt4 and β-catenin protein (K) and RNA level (L) were decreased while RNPC1 was knocked down. The same effects were also observed in BT474 cells (O, P). **Q-R**. In the absence of ER, RNPC1a regulated PR expression. ER was knocked down in MCF-7 and BT474 cells using lentivirus(sh1, sh2). Overspression of RNPC1a could enhance PR expression. The relative quantification was calculated by the ΔΔCt method and normalized based on β-actin. Data were means of three separate experiments and presented as mean ± SEM, **p< 0.01.

We chose MCF-7 and BT474 cells for further study, for PR expression of these two cell lines were neither too higher nor too lower compared with those in other PR positive breast cancer cells (Supplementary Figure S3). MCF-7 and BT474 cells were transfected with lentivirus containing either control luciferase (NC) or RNPC1a overexpression (RNPC1a). PR expression was obviously increased when RNPC1a was up-regulated both in protein and RNA levels in MCF-7 (Figure 2A and 2B, p<0.01) and BT474 (Figure 2C and 2D, p<0.01) cells. To verify endogenous RNPC1a could regulate PR expression, MCF-7 and BT474 cells were transfected with a control (SCR) and RNPC1a knockdown (shRNPC1a) lentivirus. PR protein and RNA levels in MCF-7 (Figure 2E and 2F, p < 0.01) and BT474 (Figure 2G and 2H, p <0.01) were significantly decreased. It indicated that RNPC1a could positively regulate PR expression in PR positive breast cancers.

PR could up-regulate its target gene of wingless-type MMTV integration site family, member 4 (Wnt4) and β-catenin. Wnt4 and β-catenin expression were detected in RNPC1a overexpressed and knocked down cells (Figure 2I-2P). In MCF-7 (Figure 2I-2L) and BT474 (Figure 2M-2P) cells, RNPC1a overexpressed and knocked could regulate Wnt4 and β-catenin expression both in protein and RNA level.

Considering ER could also regulate PR expression, ER was knocked down both in MCF-7 and BT474 cells. Then the MCF-7 and BT474 cells were transfected with lentivirus to overexpress or deplete RNPC1a. It was obvious that overexpression of RNPC1a still increase PR expression in MCF-7 cells (Figure 2Q) and BT474 cells (Figure 2R).

### RNPC1a regulated PR mRNA stability via binding to PR mRNA

**Figure 3:**
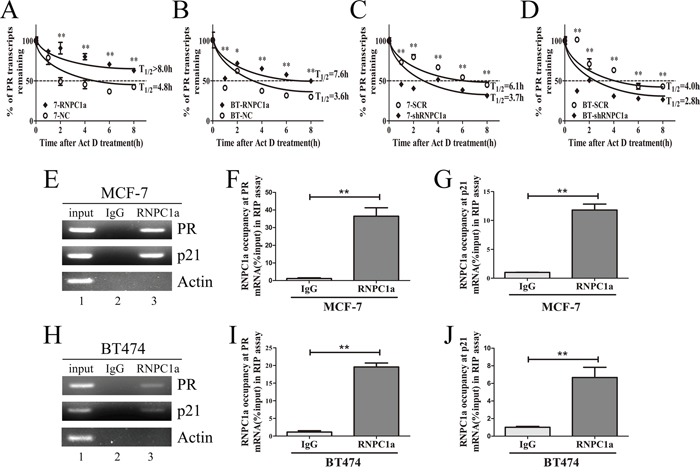
RNPC1 directly bound to PR transcript and regulated its stability **A, B**. The half-life of PR transcript was enhanced by RNPC1a overexpression. (A) MCF-7 (7) and (B) BT474 (BT) cells were transfected with lentivirus to overexpress RNPC1a. The control (NC) and RNPC1a overexpression (RNPC1a) cells were treated with 5 μg/ml actinomyclin D (Act D) for 0, 1, 2, 4, 6, 8 h. **C, D**. The half-life of PR transcript was decreased after RNPC1a knockdown. (C) MCF-7 and (D) BT474 cells were transfected with the negative control vectors (SCR) and RNPC1a knockdown lentivirus (shRNPC1a). The following experiments were conducted according to those in RNPC1a overexpression. The relative quantification was calculated by the ΔΔCt method and normalized based on β-actin. **E-J**. RNPC1a associated with PR transcript *in vivo*. (E-G) MCF-7 and (H-J) BT474 cell lysates were immunoprecipitated with RNPC1a antibody or control IgG followed by RT-PCR (E, H) and qRT-PCR (F, G, I, J) measuring transcript levels of PR, p21 within RNPC1a or IgG immunocomplexes. Upon normalization the level of actin transcript, the data were calculated from three separate experiments and performed as mean ± SEM, *p < 0.05, **p < 0.01.

The half-life of PR transcript was increased along with overexpression of RNPC1a. In MCF-7 cells, the half-life of PR transcript was increased from 4.3 h to >8 h (Figure 3A), and in BT474 cells the half-life of PR transcript was increased from 3.6 h to 7.6 h (Figure 3B), suggesting that PR mRNA stability increased after RNPC1a overexpression. Moreover, the half-life of PR transcript was decreased after RNPC1a knockdown. In MCF-7 cells, the half-life of PR transcript was decreased from 4.8 h to 3.3 h (Figure 3C). Similarly, the half-life of PR transcript was decreased from 4.0 h to 2.8 h in BT474 cells (Figure 3D). Together, these data demonstrated that RNPC1a increased the stability of PR transcript. Then, we investigated whether RNPC1a was physically associated with PR transcript. RNA immunoprecipitation assay followed by RT-PCR (Figure 3E) and qRT-PCR (Figure 3F, 3G) were performed on extracts from MCF-7 cells. It showed that PR transcript was present in RNPC1a, but not in the control IgG immune complexes (Figure 3E). p21 transcript was positive control as it had previously been described to form immune complexes with RNPC1a [27]. As negative control, β-actin mRNA was unable to bind to RNPC1a. Similarly in BT474 cells, PR, p21 transcripts were also present in RNPC1a, but not in control IgG (Figure 3H-3J). It indicated that RNPC1a could physically bind to PR transcript.

### RNPC1a bound directly to AU-rich element within the PR 3′-UTR

**Figure 4:**
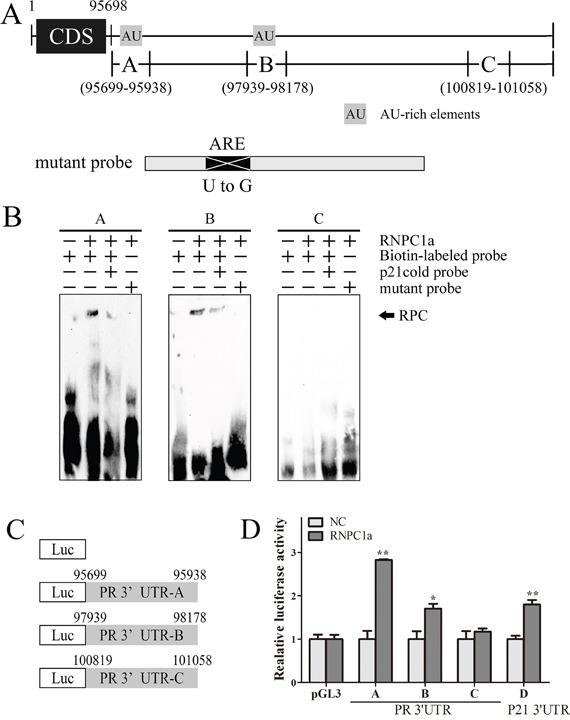
Multiple regions in the PR 3′-UTR were bound by RNPC1 **A**. Schematic representation of PR transcript and the location of probes used for REMSA. AU/U-rich regions were shown in shaded boxes. The location of mutant probe was indicated. **B**. RNPC1a bound to 3′-UTR (probe A, B, C, D). Probes A, B, D but not probes C, associated with RNPC1a. REMSA was performed by mixing probe A, B, C, or D with His-tagged RNPC1a protein, respectively. The binding of RNPC1a to p21 3′-UTR was used as competitive probe. For a competition assay, an excess amount of p21 cold probe was added to a reaction mixtu re containing RNPC1 and biotin-labeled probe. The bracket indicated RNA-protein complexes (RPC). **C**. Schematic representation of the luciferase plasmid with various region of PR 3′-UTR. **D**. The luciferase activity for the reporter carrying PR 3′-UTR-A, -B, -D and p21 3′-UTR-E was increased by RNPC1a. MCF-7 cells with RNPC1a overexpression lentivirus (RNPC1a) and the control (NC) were transfected with pGL3 reporter carrying various regions of PR 3′-UTR and p21 3′-UTR for 48 h, respectively. Cells were then harvested for luciferase assay as described in ‘Materials and methods’. The fold increase in relative luciferase activity is a product of the luciferase activity induced by RNPC1a (7-RNPC1a) divided by that induced by an empty NC (7-NC) vector.

To identify the potential binding site (s) of RNPC1a in PR transcripts, we turned our focus to 3′-UTR of PR mRNA to seek sequences potential to bind RNPC1a. Sequence analysis of PR mRNA with the UCSC Genome Browser (http://genome.ucsc.edu/) provided a series of AREs in its 3′-UTR. Furthermore a two-dimensional structure prediction algorithm (RNAfold, http://rna.tbi.univie.ac.at/cgi-bin/RNAfold.cgi) was applied to support the probability of RNPC1a to bind to these sites. Among these sites, we chose site A (probe A) and B (probe B). Besides, we chose site C (probe C) which is not rich in AU elements as the negative control (Figure 4A).

RNA electrophoretic mobility shift assay (REMSA) was used to confirm the binding site(s) of RNPC1a in PR transcript. The recombinant His-tagged RNPC1a protein formed a complex with probe A, B respectively, compared with the negative probe C (Figure 4B). Probe D from p21 3′-UTR served as competitive probe as it had previously been described to form RNA-protein complex with RNPC1a. Probe C was unable to bind to RNPC1a protein, which might be due to lack of AREs within the probe C. Besides, mutation of the RNPC1-binding site in probe A and B lead to unbinding with RNPC1a protein (Figure 4B). It suggested that RNPC1a could bind to the ARE regions of PR mRNA 3′-UTR, which could be contested by the p21 probe. To functionally confirm the ARE regions were required for RNPC1a binding to the PR transcript, we performed a dual-luciferase assay using pGL3 reporters that carried various region of PR 3′-UTR, including PR 3′-UTR -A, B, C and p21 3′-UTR -D, whose sequences were identical to probes A, B, C and D, respectively (Figure 4C). The luciferase activity for a reporter carrying PR 3′-UTR -A, B and p21 3′-UTR -D was significantly increased by RNPC1a. By contrast, the PR 3′-UTR -C were not responsive to RNPC1a (Figure 4D). Taken together, these data suggested that PR 3′-UTR -A and B were responsive to the direct binding of RNPC1a.

### RNPC1a enhanced the breast cancer cells proliferation induced by progesterone *in vitro* and *in vivo*

**Figure 5:**
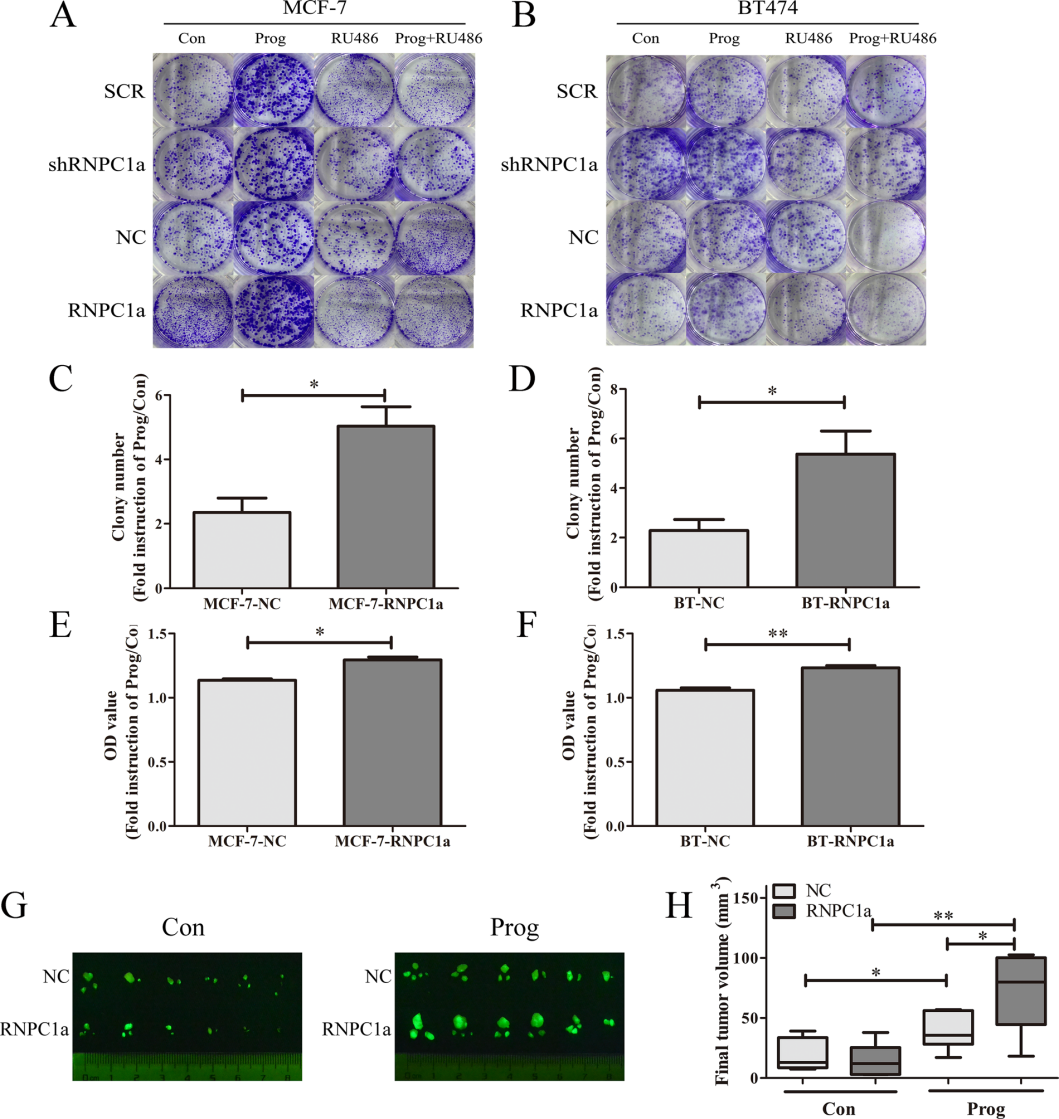
Progesterone increased breast cancer cells proliferation through PR upregulated by RNPC1a *in vitro* and *in vivo* Colony formation assay (A-D) and cell counting kit (CCK-8) assay (E, F) was conducted to investigate the proliferation of RNPC1a overexpression cells in the present of progesterone. RNPC1a was overexpressed and knockdown in MCF-7 and BT474 cells. In MCF-7 and BT474 cell lines, the RNPC1a overexpressed (RNPC1a), knockdown (shRNPC1a) and the each control cells (NC, SCR) were treated with 10 nM progesterone (Prog), 10 nM RU486 (RU486), 10 nM progesterone+10 nM RU486, or left untreated. **A-D**. The growth of cells over 20 days was measured using colony formation assays. The colony number of Prog/Con in MCF-7-RNPC1a or BT474-RNPC1a was significantly increased compared to control cells, respectively. And there seemed no difference of the effect between the two cell lines. **E, F**. The OD value of Prog/Con in overexpression MCF-7 and BT474 cells was significantly higher than that of the control cells. Data were means of three separate experiments and performed as mean ± SEM, *p < 0.05, **p < 0.01. **G, H**. Progesterone increased breast tumor formation *in vivo*. (G) Fluorescent gross of tumors growth 60 days after inoculation from the different groups of NOD/SCID mice. (H) Tumor volume was calculated, and all date were shown as Whisker: min to max, *p < 0.05, **p < 0.01.

To explore the influence of RNPC1a on PR proliferating functions *in vitro*, colony formation assay and cell counting kit (CCK-8) assay was applied (Figure 5A-5F). Overexpression of RNPC1a depressed the breast cancer cell MCF-7 and BT474 proliferation, which was in accord to our previous finding, RNPC1 as a tumor suppressor in breast cancer [28]. Progesterone could promote the proliferation of breast cancer cells. And this proliferation could be blocked by RU486, which is indicated to be a PR-dependent function (Figure 5A, 5B). This function was enhanced in RNPC1a overexpressed MCF-7 cells (Figure 5A, 5C, 5E) and BT474 (Figure 5B, 5D, 5F) cells. To further explore the influence of RNPC1a on PR proliferating functions *in vivo*, tumor formation following inoculation of MCF-7 cells in mouse models was applied. Considering MCF-7 is not high invasive breast cancer cell and its tumorigenicity is weak, human tissues was implanted in NOD/SCID mice before breast cancer cells injection. Significantly, given progesterone 5 mg for 60 days, the tumor volume in RNPC1a overexpression group was larger than that of the NC group, which might indicate that overexpression of RNPC1a up-regulated PR through which progesterone could enhance tumor proliferation *in vivo* (Figure 5G, 5H). Besides, western blot was used to verified that with or without progesterone, overexpression could upregulate PR in NOD/SCID mice tumor tissue (Supplementary Figure S4).

### RNPC1a enhanced mitogen-activated protein kinase (MAPK) activation induced by progesterone *in vitro*

**Figure 6:**
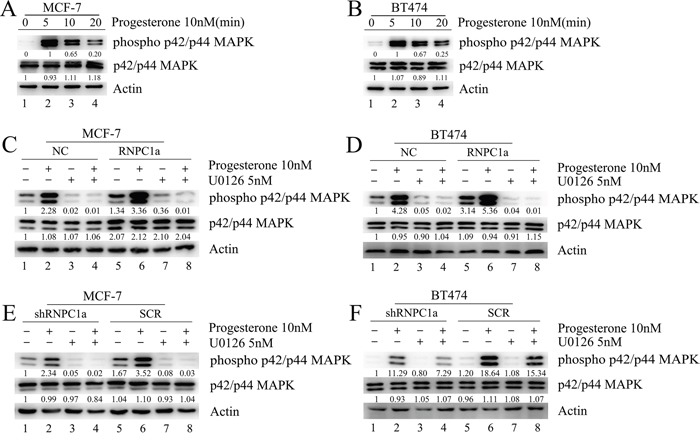
Progesterone induced phospho-p42/p44MAPK activation was enhanced by RNPC1a in MCF-7 and BT474 cells **A, B**. Activation of phospho-p42/p44 MAPK in MCF-7 and BT474 cells treated with progesterone. **C, D**. MCF-7 and BT474 cells transfected with lentivirus to overexpress RNPC1a (RNPC1a) and the control (NC) were left untreated or were treated for the indicated times with progesterone (10 nM) in the absence or presence of the selective MAPK inhibitor U0126 (5 nM). Progesterone activated MAPK pathway rapidly, which impact was more obviously on RNPC1a (C, lane 6) than on NC (C, lane 2) and could be inhibited by U0126 (C, lane 3, 7). The same effects were also observed in BT474 cells (D). **E, F**. MCF-7 and BT474 cells transfected with lentivirus to knockdown RNPC1a (shRNPC1a) and the control (SCR) were left untreated or were treated for the indicated times with progesterone (10 nM) in the absence or presence of the U0126 (5 nM). MCF-7 cells transfected with lentivirus to knockdown RNPC1a (shRNPC1a) and be the control (SCR) (6E). The effect of progesterone on MAPK activation was decreased after RNPC1a knockdown (6E, lane 2, 6), which could be inhibited by U0126 (E, lane 3, 7). These effects were also observed in BT474 cells (F). Besides all of these results, overexpression of RNPC1 indeed increased the expression of total p42/p44 MAPK, which was obvious as RNPC1was overexpression in MCF-7 cells (Figure 6C). Westeren bolt results in Figure 6 were quantitated. These experiments were repeated three times with the same results.

Progesterone activated MAPK pathway rapidly in MCF-7 and BT474 cells (Figure 6A, 6B). MCF-7 cells were transfected with lentivirus containing either control luciferase (NC) or RNPC1a overexpression (RNPC1a) (Figure 6C). Considering the protein expression of PR needed a relative long time for about 20 h. In this experiment we detected the protein expression excluding PR only 5 min after dealing with drugs. Progesterone activated MAPK pathway rapidly. This effect was more obviously on RNPC1a (Figure 6C, lane 6) than on NC (Figure 6C, lane 2) and could be inhibited by the inhibitor of MAPK kinase U0126 (Figure 6C, lane 3, 7). This effect was also observed in BT474 cells (Figure 6D). MCF-7 cells were transfected with lentivirus to knockdown RNPC1a (shRNPC1a) or with the control (SCR) (Figure 6E). The effect of progesterone on MAPK activation was decreased after RNPC1a knockdown (Figure 6E, lane 2, 6), which could be inhibited by U0126 (Figure 6E, lane 3, 7). The same effect was also observed in BT474 cells (Figure 6F).

## DISCUSSION

In the present study, we described a unique mechanism that PR expression could be regulated by RNPC1, a RNA-bind protein via stabilizing its mRNA.

Our previous study found that RNPC1 expression differed between normal breast tissue and breast cancer tissue, which was significantly associated with PR status in breast cancer patients [28]. Herein, we confirmed the relationship between RNPC1 and PR by IHC analysis. Moreover, overexpression of RNPC1 increased, whereas knockdown of RNPC1 decreased the levels of PR protein and transcript in PR positive breast cancer cells. RNPC1, emerging as a target of p53 family, could extensively regulate a range of genes including CG33336 gene product from transcript CG33336-RB (p53), tumor protein p63 (p63), tumor protein p73 (p73), cyclin dependent kinase inhibitor 1A (p21), ELAV like RNA binding protein 1 (HuR), and MDM2 proto-oncogene (MDM2). Among these regulation, RNPC1 could directly bind to AREs within 3′-UTR of target genes mRNA, and adjust their mRNA stability, and exert influence on their relevant functions [27, 29, 30, 31, 32]. In the present study, we also found that RNPC1 could change the stability of PR transcript and eventually increase its half-life. Moreover, we confirmed that RNPC1 directly bound to the two AREs regions within 3′-UTR of PR transcript by REMSA and dual-luciferase reporter assay. So it was concluded that RNPC1 could bind directly to the AREs regions within 3′-UTR of PR and stabilize PR mRNA and regulate the PR expression in PR positive breast cancer.

Considering PR serving as a progesterone-dependent nuclear receptor transcription factor, progesterone was employed to verify the functions of PR regulated by RNPC1. Progesterone could elicit PR-dependent cell proliferation in the manners of autocrine/paracrine signaling and transcriptional regulation [33]. In addition, previous studies proved that progesterone could induce nongenomic activation of p42/p44 MAPKs in breast cancer through the classical PR pathway and consequently result in cell proliferation [34]. *In vitro* experiment found that progesterone could enhance the proliferation of breast cancer cells [35]. Moreover, this effect was increased as RNPC1 was overexpressed. Meanwhile, the progesterone could rapidly activate the MAPK cascade within 5 min, which could be accelerated by RNPC1 overexpression in breast cancer cells. The enhancing progesterone-dependent PR functions and following breast cancer cells proliferation by RNPC1 were due to the PR up-regulation by RNPC1 via stabilizing the PR mRNA.

To verify the functions of PR regulated by RNCP1 *in vivo*, we implanted human breast tissue in NOD/SCID mice with bilateral ovariectomy, which could provide humanized breast microenvironment [36]. The human tissue-specific microenvironment is a fundamental factor in orthotopic and metastatic breast cancer mouse models of clinically relevant studies which could improve efficiency and stability of breast cancer xenografis formation [36]. Bilateral ovariectomy was aimed to eliminate the effect of endogenous ovaries hormone. In the ovariectomized NOD/SCID mice, progesterone had significantly affect breast tumor formation. Distinctly, the tumor formation with RNPC1 overexpression stimulated by progesterone was increased more markedly. This *in vivo* result was in accordance to the observation *in vitro*, confirming the enhancing progesterone-dependent PR functions were due to the PR up-regulation by RNPC1 via stabilizing the PR mRNA.

In summary, the present study revealed a unique role for RNPC1 in the regulation of PR and its functions. Importantly, we provided a novel PR regulating mechanism by modifying mRNA stability. Our previous study found that RNPC1 could also regulate ER expression through stabilizing its mRNA [37]. Considering the significance of ER and PR for the classification and therapy of breast cancer, implied that RNPC1 might be a potential molecular mark for diagnosis and therapy of breast cancer, especially in adjuvant endocrine therapy of breast cancer.

## MATERIALS AND METHODS

### Cell culture, treatment and transfection

The human breast cancer cell lines MCF-7 and BT474 were obtained from American Type Culture Collection (ATCC, USA) and culture in complete medium of High glucose Dulbecco's Modified Eagle Medium (DMEM) supplemented with 10% fetal bovine serum (FBS), 1% penicillin-streptomycin solution at 5% CO_2_ and 37°C incubator.

For progesterone or RU486 treatment, MCF-7 and BT474 cells were cultured in DMEM without phenol red, supplemented with 5% steroid-depleted fetal bovine serum (BI, Israel) for 2 days before progesterone (Sigma, USA) or RU486 (Sigma, USA) treatment. To block p42/p44MAPK activation, 5 nM U0126 (Sigma, USA), dissolved in 1:2000 dimethyl sulfoxide (DMSO), was added to wells 90 min before incubation with progesterone.

Lentivirus constructs of RNPC1a overexpression and knockdown were generated as previously described [37]. The breast cancer cells were stably transfected with RNPC1a overexpression lentivirus (termed as RNPC1a), RNPC1a knockdown lentivirus (termed as sh1, sh2, sh3), a negative control (termed as NC) and a scramble control (termed as SCR). In our earlier studies, three different shRNA were used to knock down RNPC1, but only one of those work well. So we chose the most effective shRNA for the further experiment. Cells were plated in 6 wells dishes at 30%-40% confluence and infected with the retroviruses. Simultaneously, polybrene (5 μg/ml) was added with the retroviruses to improve infection efficiency. Stable pooled populations of breast cancer cells were generated by selection using puromycin (3 μg/ml) for 2 weeks. For RNPC1a knockdown, one construct (sh2) named as shRNPC1a, with ≥85% knockdown efficiency was used for further studies [37]. Lentivirus constructs of ERαknockdown were generated as RNPC1a described before.

### Western blotting analysis

Protein isolation was acquired with total protein isolation kit (KeyGen, Nanjing, China). The total proteins were then electrophoresed by 10% SDS-PAGE gel and transferred to polyvinylidene fluoride (PVDF, Roche, Switzerland) membranes. The blots were probed or reprobed with antibodies. The membranes were probed using Immobilon Western Chemiluminescent HRP Substrate (Millipore, USA) and autoradiographed. The primary antibodies used were anti-rabbit RBM38, the alia name of RNPC1, (Santa Cruz, USA), PR (Cell Signaling technology, USA), total p42/p44 MAPK (Cell Signaling technology, USA), phospho p42/p44 MAPK (Cell Signaling technology, USA), Wnt (Cell Signaling technology, USA), β-catenin (Cell Signaling technology, USA) anti-mouse β-actin (Cell Signaling technology, USA). The anti-rabbit and anti-mouse secondary antibodies were purchased from Cell Signaling technology (USA). β-actin was used to normalize protein loading. The level of protein was quantified by densitometry.

### RNA extraction, reverse transcription and quantitative RT-PCR (qRT-PCR)

Total RNA was extracted with Trizol reagent (TaKaRa, Japan), and cDNA was synthesized using Primescript RT Reagent (TaKaRa, Japan) following manufacturer's instructions. The PCR program used for amplification was (i) 94°C for 30 s, (ii) 94°C for 30 s, (iii) 55°C for 30 s, (iv) 72°C for 1 min, and (v) 72°C for 10 min. From steps 2 to 4, the circulation was repeated 35 times for β-actin and other genes. The following PCR primers were used: RNPC1a: Forward, 5′-ACGCCTCGCTCAGGA AGTA-3′ RNPC1a: Reverse, 5′-GTCTTTGCAA GCCCTCT CAG-3′ β-actin: Forward, 5′-GCTGTG CTATCCCTGTAC GC-3′ β-actin: Reverse, 5′-TGCCTC AGGGCAGCGGAA CC-3′ PR: Forward, 5′- ACCCG CCCTATCTCAACTACC -3′ PR: Reverse, 5′- AGGACA CCATAATGACAGCCT -3′ p21: Forward, 5′-TGTCCG TCAGAACCCATGC-3′ p21: Reverse, 5′-AAAGTC GAAGTTCCATCGC TC- 3′ Wnt4: Forward, 5′- AGG AGGAGACGTGCGAGAAA -3′ Wnt4: Reverse, 5′- CGA GTCCATGACTTCCAGGT - 3′ β-cateinin: Forward, 5′- CCTATGCAGGGGTGGTCAAC-3′ β-cateinin: Reverse, 5′- CGACCTGGAAAACGCCATCA-3′ All PCR reactions were performed using the fluorescent SYBR Green I methodology. Quantitative RT-PCR (qRT-PCR) was performed on StepOnePlus Real-Time PCR system (Applied Biosystems, USA) with Fast Start Universal SYBR Green Master (Roche, Switzerland) according to the manufacturer's instructions. The relative quantification was calculated by the 2^-ΔΔCt^method.

### Immunofluorescence (IF)

The expression location of RNPC1a and PR was conducted by the immunofluorescence, as previously described [37]. Briefly, the breast cancer cells were plated in 24-well plate at the density of 5×10^4^ cells per well. After 36 h incubation, the cells were washed with phosphate-buffered saline (PBS, pH 7.4) twice, and then fixed with paraformaldehyde for 20 min and penetrated by 0.5% Tritonx-100 for 10 min, followed by blocking for 1 h in blocking buffer. Then the cells were incubated with primary antibody at 4°C overnight. After washed with PBS three times, the cells were incubated for 1 h in the dark with FITC-conjugated secondary goat anti-rabbit antibodies (Invitrogen, USA). The cells were then washed and stained with 4, 6-diamidino-2-phenylindole (DAPI) for 5 min. Immunostaining was observed under a Zeiss fluorescence microscope at 400× magnification.

### CCK-8 assay

Cell proliferation was assessed by using CCK-8 kit (Dojindo, Japan) following the manufacturer's instruction. Briefly, 5×10^3^ cells were seeded into a 96-well plate. After12 h incubation, cells were incubated in the presence or absence of 10 nM progesterone. On the days of measuring the growth rate of cells, the medium in each well was replaced with 100 μl fresh medium containing 10% CCK-8. The plates were incubated at 37°C for 2 h and then read at 450 nm with a microplate reader (Groding, Tecan, Austria). All tests were performed in triplicate.

### Colony formation assay

Cells used for colony formation analysis was conducted as previously described [28], The breast cancer cells were seeded into 6-well plates (1000 cells/well). 12 h later, cells were incubated in the presence or absence of 10 nM progesterone and 10 nM RU486 for 20 days. The formatting colonies were fixed in paraform and stained with Giemsa (Sigma, USA) after washed by PBS twice, then dried at room temperature.

### IHC staining

The breast cancer sample tissue microarrays (BC08118) for IHC analysis were purchased from Biomax (USA). Histologic types were classified according to the World Health Organization (2003). TNM staging was defined according to the American Joint Committee on Cancer (AJCC) (the 6th version, 2002). The IHC staining was performed as previously described [38, 39]. The same tissue samples were stained with RNPC1a and PR antibody respectively. The RBM38 antibody (LifeSpan Biosciences, USA) was used at the dilution of 1:350. The PR antibody (Cell Signaling technology, USA) was used at the dilution of 1:500. The rabbit polyclonal antibody was used as anti-RBM38 and PR primary antibody. The breast cancer tissues were scored by semiquantitative analysis upon a well-established immunoreactivity scoring system (IRS) [40]. The final staining results of RNPC1a and PR were described as follows. The staining intensity (SI) was scored on a scale of 0-3. The score 0 was attained for totally negative cases. For weak, moderate, and strong staining, the scores were 1, 2 and 3, respectively. Secondly, the percentage of positive cells (PP) was scored into five categories: no staining, 1-10, 11-50, 51-80, 81-100 percentage positive cells. And the scores were 0, 1, 2, 3 and 4, respectively. An IRS was calculated by multiplying the percentage of PP times the SI score, resulting in a scale from 0 to 12. The IRS was divided into three groups: negative (IRS 0-3), or low staining (IRS 4-7) and high staining (IRS 8-12). The tissue microarrays were observed under 200 × magnifications. All the cases were individually categorized by two independent pathologists.

### RNA immunoprecipitation (RIP)

RIP was carried out as previously described. Briefly, the breast cancer cells (2×10^7^) were lysed with RNA immunoprecipitation lysis buffer (Millipore, USA) and then incubated with 5 μg of rabbit polyclonal anti-RBM38 or non-immunized rabbit IgG at 4°C overnight. The RNA-protein immunocomplexes were brought down by protein A/G magnetic beads, followed by RNA purification. After that, the purified RNA was subjected to RT-PCR and qRT-PCR.

### REMSA

*E.coli*BL21 (DE3) was transformed with a pET28a vector expressing His-tagged RNPC1a and positive clones were selected. After induction by isopropyl β-D- 1-thiogalactopyranoside (IPTG), the recombinant proteins were then purified by Ni-NTA beads (GE Healthcare, UK), as describe in previous study [37].

The UCSC Genome Browser (http://genome.ucsc.edu/) and a two-dimensional structure prediction algorithm (RNAfold, http://rna.tbi.univie.ac.at/cgi-bin/RNAfold.cgi) were used to filtrate the potential ARE sites of PR mRNA 3′-UTR.

To generate REMSA probes, various regions in PR (A-C) and p21(D) 3′-UTR were PCR-amplified using primers containing T7 promoter sequence (5′- TAATACGACTCACTATAGGG -3′). The sequences of PCR product were listed in Table 1S. Mutant probes were mutated in AREs with U to G.

RNA probes were made from *in vitro* transcription with a MEGA shortscript Kit (Ambion, USA) in the presence of biotin-16-UTP (Roche, Switzerland) following the manufacturer's instruction.

REMSA was performed with a LightShift Chemiluminescent RNA EMSA Kit (Thermo, USA) following the manufacturer's instruction. Briefly, 4 mg/ml purified RNPC1, 10 mg/ml of tRNA, 2 nM biotin-labeled RNA probe were mixed in a REMSA binding buffer (10 mM HEPES (pH 7.3), 20 mM KCl, 1 mM MgCl_2_, 1 mM dithiothreitol) and incubated for 30 min at room temperature. RNA/protein complexes were then electrophoreticed by 4% native polyacrylamide gel and transferred to nylon membrane (Thermo, USA). RNA was cross-linked with a UV lamp at a distance of 0.5 cm from the membrane for 3 min. The membrane was blocked in blocking buffer for 15 min and replaced the blocking buffer with conjugate/blocking buffer (stabilized streptavidin-horseradish peroxidase conjugate 1:300 dilution). After washed with 1× wash buffer for 3 times, membrane was incubated in substrate equilibration buffer for 5 min. Then, the membrane was incubated in substrate working solution and exposed.

### Dual-luciferase reporter assay

Dual-luciferase reporter assay was performed in triplicate according to manufacturer′s instructions (Promega, USA). Briefly, 5 ng of Renilla luciferase vector (pRL-CMV; Promega, USA), an internal control, and 200 ng of a pGL3 reporter which contained various region of PR and p21 3′-UTR were co-transfected into MCF-7 and BT474 RNPC1a overexpression (RNPC1a) and the control (NC) cells. 48 h after transfection, luciferase activity was measured with the Dual-Luciferase reporter assay system (Promega, USA) according to manufacturer's procedure. The fold change in relative luciferase activity is a ratio of the luciferase activity induced by RNPC1a divided by that induced by NC.

### Human tissues implantation and bilateral ovariectomy

Four- to five-weeks old female non obese diabetic/severe combined immunodeficient (NOD/SCID) mice were purchased from Model Animal Research Center of Nanjing University (MARC, Nanjing, China). NOD/SCID mice were kept under specific pathogen free (SPF), temperature-controlled conditions (20-24°C, humidity of 40-70%). Cages, bedding, and drinking water were autoclaved and changed regularly. Food was sterilized by irradiation. The mice were maintained on a daily cycle of 12 h of light and 12 h of darkness.

Normal human breast tissues were obtained from freshly discarded material of elective breast reduction mammoplasty surgery. Sample collection was performed in accordance with the ethical guidelines of the Declaration of Helsinki, and approved by the ethics and research committee of the First Affiliated Hospital of Nanjing Medical University. Breast tissues were stripped of excess fat and sliced under sterile conditions into pieces ∼4 mm in width. Three pieces were selected randomly for histological examination to exclude primary malignant disease. Tissues were placed in ice cold PBS until implantation into NOD/SCID mice. Implantation was finished within 6 h after removal surgery. Prior to implantation, mice were anesthetized by intraperitoneal injection with 1% pentobarbital sodium (10 μl/g body weight) (Sigma, USA). Surgical procedures were performed as previously described, with some modifications [25]. Briefly, 5-6 mm scalpel incisions were made in the skin of the mid-dorsal flank, through which three pieces of human breast tissue were implanted subcutaneously. The mice received gentamycin in the drinking water (800,000 U/L) up to one week following the implantation. In the model, human breast tissue was implanted in both the left and the right mid-dorsal flanks. In the meanwhile, bilateral ovaries of NOD/SCID mice were removed under anesthesia.

### Injection of breast cancer cells and implantation of progesterone pellets into NOD/SCID mice

One week after human tissues implantation and bilateral ovariectomy, MCF-7-NC and MCF-7-RNPC1a cells grown to about 80% confluence were harvested using 0.25% trypsin and 0.02% disodium EDTA, washed in media, counted, and re-suspended in PBS. 1×10^6^ cells in 0.2 ml PBS of MCF-7-NC and MCF-7-RNPC1a cells were injected into the bilateral human breast tissue in NOD/SCID mice. Then mice injected with MCF-7-NC or MCF-7-RNPC1a cells were randomly divided into two groups respectively: control group (Con, n=6) with no treatment and progesterone group (Prog, n=6) implanted with 60-day slow-release progesterone pellets (5 mg, Innovative Research of America) each mouse.

### Gross observation of tumor under fluorescence

60 days after inoculation of the breast cancer cells, all mice were sacrificed and observed grossly by the Whole Body Imaging System (Illumatool 9900, Lightools Research, USA). Tumors with green fluorescence were harvested and checked for final tumor size with external calipers.

### Statistical analysis

The data were analyzed using the SPSS 20.0 software. All experiments in this study were repeated in triplicate, unless otherwise specified. The χ^2^ test was used to assess the correlation between RNPC1a and the clinic pathological parameters. The linear correlation analysis was used to assess the correlation between RNPC1a and PR.

The data of mice tumor volume were shown as Whisker: min to max. The other data were presented as mean ± SEM. For all the continuous variables, Student t-test was used to analyze the statistical significance of the differences between groups, and P<0.05 was considered to indicate a statistically significant difference.

## SUPPLEMENTARY FIGURES AND TABLES




