# Supplementary material for: RNPC1 enhances progesterone receptor functions by regulating its mRNA stability in breast cancer
Source: Oncotarget. 2016 Sep 14;8(10):16387–400. doi: 10.18632/oncotarget.12016 (PMC5369970; doi:10.18632/oncotarget.12016)
Supplement: Supplementary file 1 [file oncotarget-08-16387-s001.pdf]

RNPC1 enhances progesterone receptor functions by regulating its mRNA stability in breast cancer

SUPPLEMENTARY FIGURES AND TABLES

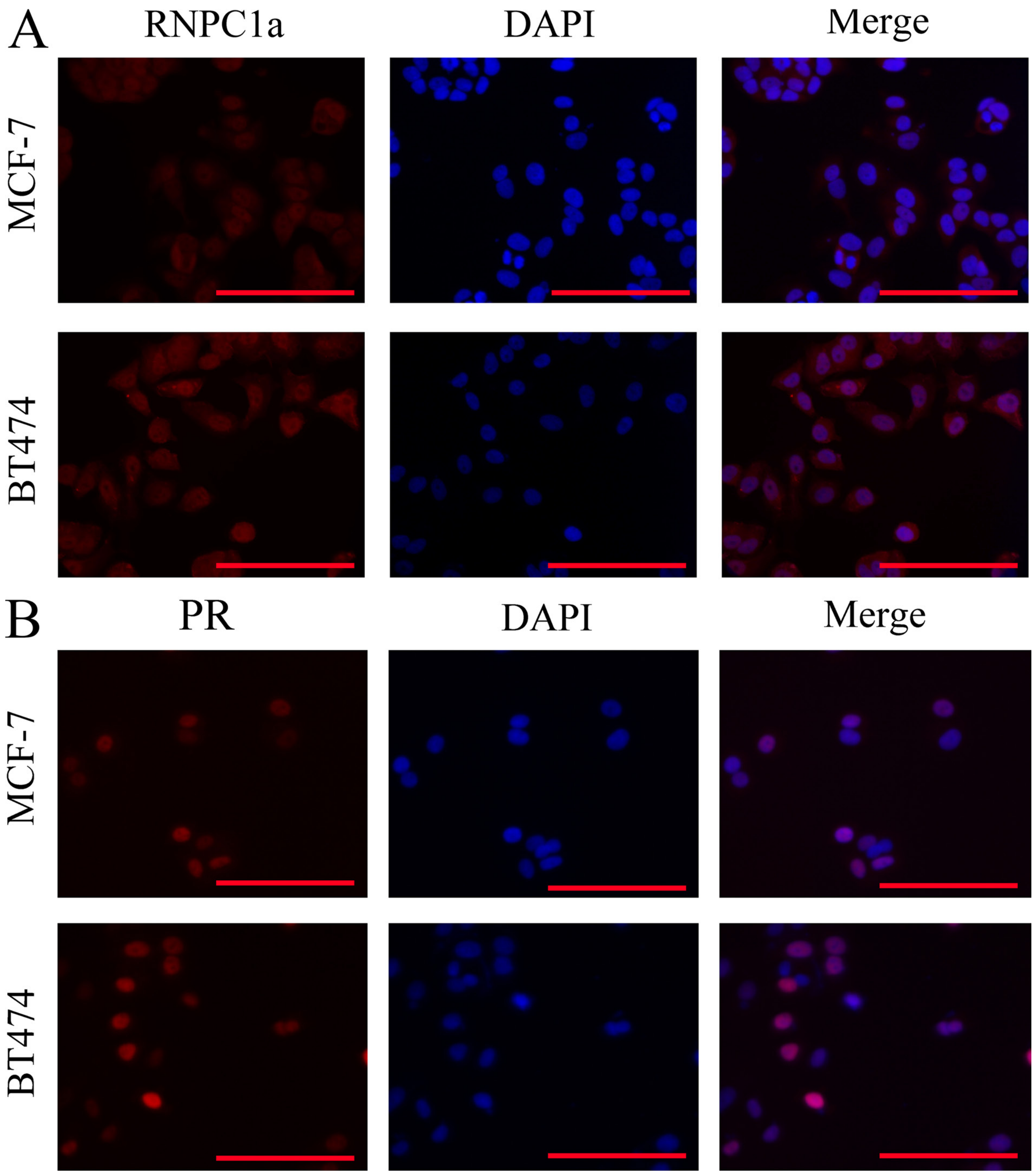

Supplementary Figure S1: RNPC1a and PR cellular localization in breast cancer cells with immunofluorescence. A. In MCF-7 and BT474 cells, RNPC1a was mainly expressed in the nucleus and cytoplasm. **B.** PR was mainly expressed in the nucleus in MCF-7 and BT474 cells.

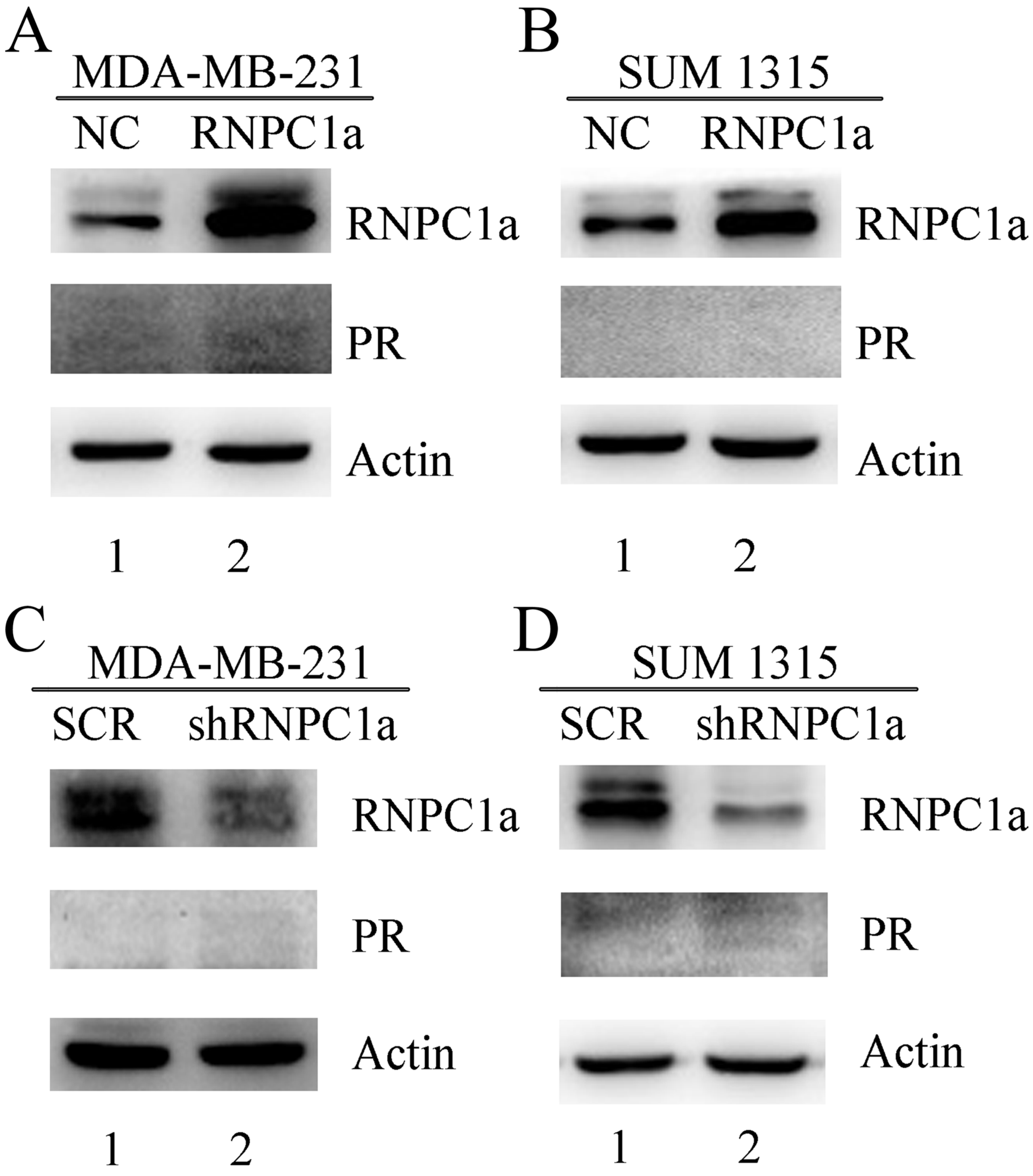

Supplementary Figure S2: RNPC1 couldn't affect PR expression in PR negative breast cancer cells. A, B. The expression of PR was not influenced by RNPC1a overexpression in PR negative breast cancers. MDA-MB-231 and SUM 1315 were transfected with lentivirus containing either control luciferase (NC) or RNPC1a overexpression (RNPC1a). Western blot was used to analyze the expression of RNPC1a and PR. **C, D.** The expression of PR was not influenced by RNPC1a knockdown. MDA-MB-231 and SUM 1315 were transfected with a control (SCR) and RNPC1a knockdown (shRNPC1a) lentivirus. Western blot was used to analyze the expression of RNPC1a and PR.

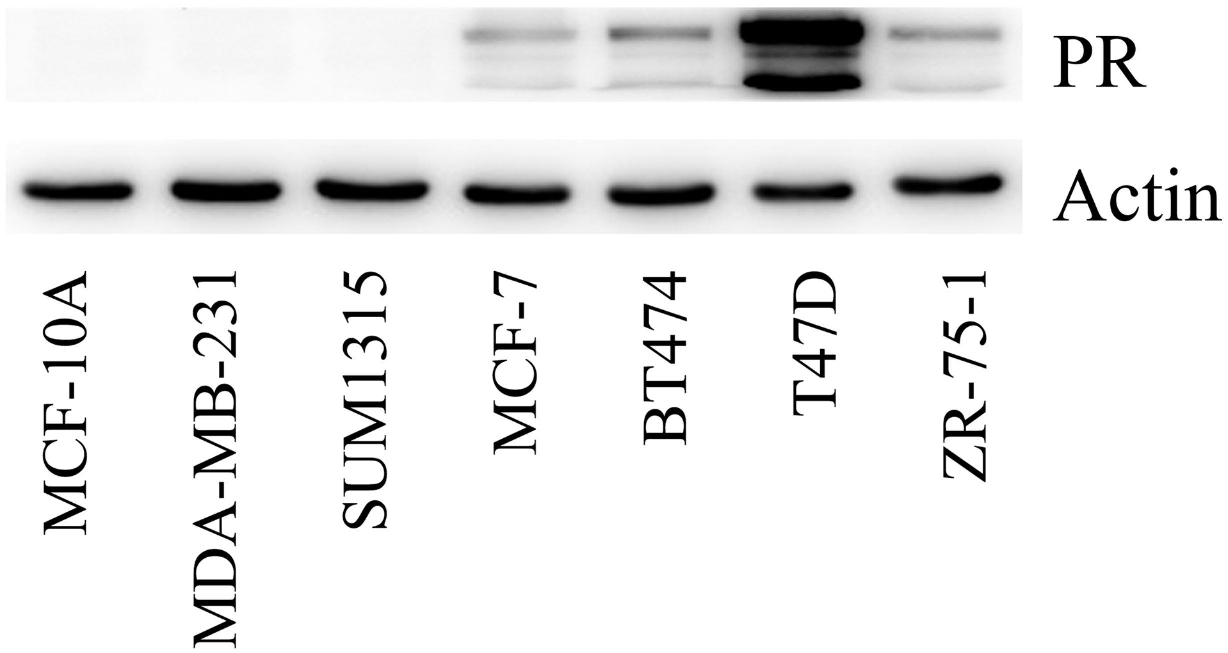

Supplementary Figure S3: PR expression in different cell lines. Western blot was used to detect PR expression in different cell lines. Normal breast cell MCF-10A and breast cancer cells MDA-MB-231 and SUM 1315 were PR negative. Breast cancer cells MCF-7, BT474, T47D and ZR-75-1 were PR positive. Among these PR positive cells, we chose MCF-7 and BT474 in our study, both of which expressed PR neither too higher nor too lower.

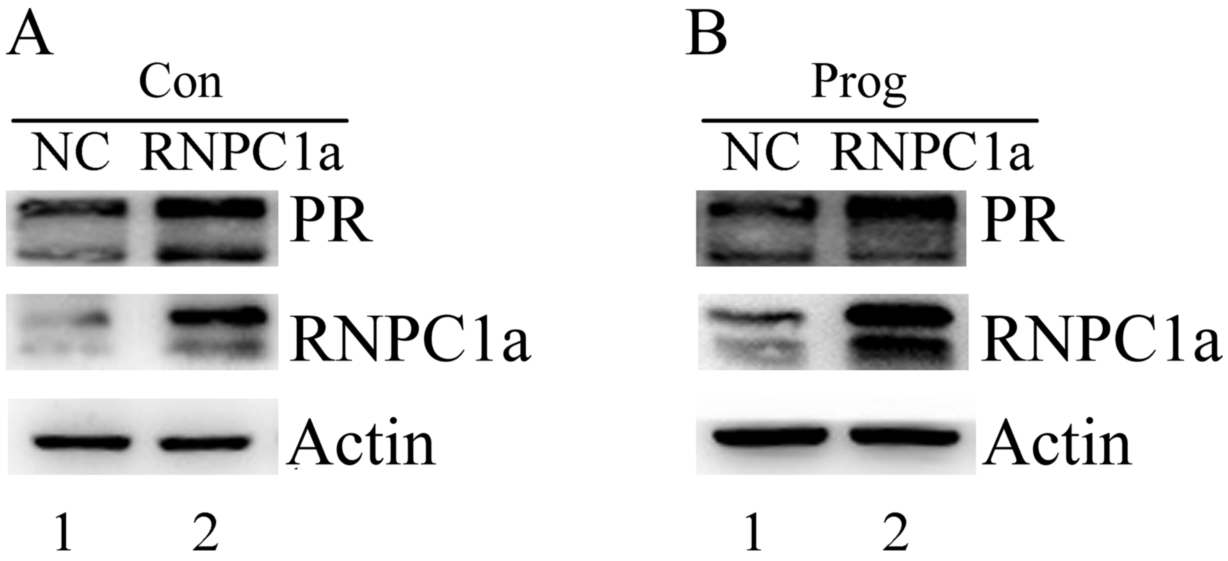

Supplementary Figure S4: RNPC1a and PR expression in NOD/SCID mice tumor tissue. A. Without progesterone, RNPC1a and PR expression in NOD/SCID mice tumor tissue was both upregulated as RNPC1a was overexpression. **B.** With progesterone, RNPC1a and PR expression in NOD/SCID mice tumor tissue was also both upregulated as RNPC1a was overexpression.

Supplementary Table S1: Sequence of REMSA probes

See Supplementary File: 1
