# Supplementary material for: RNPC1 enhances progesterone receptor functions by regulating its mRNA stability in breast cancer
Source: Oncotarget. 2016 Sep 14;8(10):16387–400. doi: 10.18632/oncotarget.12016 (PMC5369970; doi:10.18632/oncotarget.12016)
Supplement: Supplementary file 2 [file oncotarget-08-16387-s002.docx]

**Table 1: Sequence of REMSA probes**

| Probe | Sequence |
| --- | --- |
| A | TAATACGACTCACTATAGGGATGTCATCTTTTTCTTTTAAAGAATTAAATTTTGTGGTATGTCTTTTTGTTTTGGTCAGGATTATGAGGTCTTGAGTTTTTATAATGTTCTTCTGAAAGCCTTACATTTATAACATCATAGTGTGTAAATTTAAAAGAAAAATTGTGAGGTTCTAATTATTTTCTTTTATAAAGTATAATTAGAATGTTTAACTGTTTTGTTTACCCATATTTTCTTGAAGAATTTACAAGATTGAAAAA |
| B | TAATACGACTCACTATAGGGTTTGAATTAAACCAATATTTTGATGATATAAATCATTTCCACCAGCATATATTTAATTTCCATAATAACTTTAAAATTTTCTAATTTCACTCAACTATGAGGGAATAGAATGTGGTGGCCACAGGTTTGGCTTTTGTTAAAATGTTTGATATCTTCGATGTTGATCTCTGTCTGCAATGTAGATGTCTAAACACTAGGATTTAATATTTAAGGCTAAGCTTTAAAAATAAAGTACCTTTT |
| C | TAATACGACTCACTATAGGGTTAGGAATTAGCAGAACGTGCGTGGTGAGGAGATGCCAAAGGCAAGAAGAGAAGAGTATTCCAAACAGGAGGGATTCCAAAGAGAGAAGAGTATCCCAAACAACATTTGCACAAACCTGATGGGGAGAGAGAATGTGGGGTGGGGATGGATGATGAGACTGAAGAAGAAAGCCAGGTCTAGATAATCAGTGGCCTTGTACACCATGTTAAAGAGTGTAGACTTGATTCTGTTGTAAACAG |
| D | TAATACGACTCACTATAGGGTCTTAATTATTATTTGTGTTTTAATTTAAACACCTCCTCATGTACATACCCTGGCCGCCCCCTGCCCCCCAGCCTCTGGCATTAGAATTATTTAAACAAAAACTAGGCGGTTGAATGAGAGGTTCCTAAG |
